# Chondrocalcinosis of Femoro-Tibial and Proximal Tibio-Fibular Joints in Cadaveric Specimens: A High-Resolution CT Imaging Study of the Calcification Distribution

**DOI:** 10.1371/journal.pone.0054955

**Published:** 2013-01-25

**Authors:** Sébastien Touraine, Hang Korng Ea, Valérie Bousson, Martine Cohen-Solal, Liess Laouisset, Christine Chappard, Frédéric Lioté, Jean-Denis Laredo

**Affiliations:** 1 Radiologie Ostéo-articulaire, Hôpital Lariboisière, CNRS-UMR 7052, Laboratoire B2OA, Université Paris-Diderot, Sorbonne Paris Cité, Paris, France; 2 Rhumatologie, Hôpital Lariboisière, Inserm-UMR 606, Université Paris Diderot, Sorbonne Paris Cité, Paris, France; University of Notre Dame, United States of America

## Abstract

**Objectives:**

To analyze calcium deposits by computed tomography (CT) in femoro-tibial compartments and proximal tibio-fibular joints; to assess the relationship with CT-assessed osteoarthritis (OA).

**Methods:**

68 (34 pairs) cadaveric knees (mean age of 84) were scanned at high resolution CT. Menisci and hyaline cartilage calcifications in the femoro-tibial and proximal tibio-fibular joints were analyzed. OA was CT-assessed by the Kellgren and Lawrence score. Gross appearance of OA was evaluated on 29 left knees after dissection and India ink staining of tibial plateaus.

**Results:**

In femoro-tibial joints, meniscal calcifications (MC) and hyaline cartilage calcifications (HCC) were detected in 23(34%) and 14(21%) knees respectively. Calcifications mainly involved the three meniscal segments and were mainly observed in all thirds of the femoro-tibial compartments. In proximal tibio-fibular joints, HCC were detected in 19(28%) knees. The association HCC-MC in femoro-tibial joints and between calcifications in femoro-tibial and proximal tibio-fibular joints was strong (p<0.0001). Femoro-tibial and proximal tibio-fibular CT-assessed OA were respectively found in 23(34%) and 19(28%) knees. HCC were significantly associated with femoro-tibial OA (p = 0.04) while MC were not (p = 0.34). OA macroscopic evaluation showed a mean surface of cartilage lesions of 35% (range 0.13–0.55). No significant difference was demonstrated regarding the CT-detection of MC, HCC or CT-assessed OA.

**Conclusions:**

This is the first study to report a strong association of chondrocalcinosis between femoro-tibial and tibio-fibular joints in addition to a strong association between MC and HCC in femoro-tibial compartments. No significant relationship between chondrocalcinosis and OA was demonstrated.

## Introduction

Since the report by Zitnan and Sitaj in 1963 [1], chondrocalcinosis has been identified as a well-recognized but complex entity. In 2011, the EUropean League Against Rheumatism (EULAR) defined chondrocalcinosis as cartilage calcifications mainly linked to calcium pyrophosphate (CPP) crystals and detected by imaging or histological examination [2]. Nevertheless, coexistence of other calcium-containing crystals has been fully recognized [3]. The significance of articular cartilage mineralization by calcium crystals remains controversial considering the composition of calcium-containing crystals, clinical symptoms and onset of osteoarthritis (OA). However, there are growing evidences supporting the hypothesis of a pathogenic role of articular calcifications in OA. Several human studies have recently demonstrated the presence of basic calcium phosphate (BCP) crystals in patients with severe knee and hip OA [4], [5]. Fuerst et al. recently pointed the significant association between BCP detection by digital radiographs, their characterization using Fourier Transformed InfraRed (FTIR) spectroscopy and cartilage damage severity while CPP crystals were not correlated with cartilage breakdown [4]–[6]. Many studies have confirmed the noxious role of calcium-containing crystals in cartilage degradation and synovial inflammation [7]–[9]. There is growing evidence for a “crystal-induced stress” leading to the production or deposition of inorganic calcium phosphates (Pi) or pyrophosphates (PPi) in the cartilage or fibrocartilage [10]. It may also represent new therapeutic perspectives targeting calcium crystals in OA [11]–[14]. Using FTIR, Nguyen et al. observed that cartilage calcifications were diffuse in the knee joint and involved all articular compartments, including non-weight-bearing sites, which suggests that the cartilage mineralization process could reflect a generalized chondrocyte dysfunction [15]. Therefore, we proposed to study at computed tomography (CT) the distribution of articular cartilage calcifications in femoro-tibial and proximal tibio-fibular joints. Since knees are a key-target for both chondrocalcinosis and OA, we also studied the association between chondrocalcinosis and OA, assessing the gross appearance of the cartilage after knee dissection and ink staining.

## Materials and Methods

### Ethics Statement

This work complied with the Helsinki Declaration related to research carried out with human subjects. Permission to perform an imaging study on cadaveric knee specimens was obtained from the institutional review board (*Institut d’Anatomie, unité de formation et de recherche biomédicale des Saints-Pères, université Paris V*). Sixty-eight (34 pairs) of non-embalmed cadaveric knees derived from subjects who anonymously willed their body to Science, were imaged using a clinical CT-scanner.

### Cadaveric Knees

There were 34 subjects, 20 women and 14 men with a mean age of 84 years at death (range 58–96, standard deviation 9.7). None of the specimens had clinical or radiographic evidence of tumoral disease, trauma or surgical intervention. No further details were provided regarding the patient previous medical or family history. The specimens were kept at −20°C. There were thawed at room temperature and placed in a mold of paperboard and plaster in the neutral position for CT examination.

### CT Scans

All CT scans were performed under the same standardized conditions. A 32-detector row CT scanner (Somatom Sensation, Siemens, Erlangen, Germany) was used for transverse spiral imaging with 130 mm scan length centered on the femoro-tibial joint. The following protocol was used: Ultra High Resolution mode, 120 kV, 250 mAs, time collimation of 0.3 mm increments, high-resolution B40s and B70s filters, 130 mm field of view and 512×512 matrix. Multiplanar reformations with 0.5 mm sections and no gap were performed in the three reference planes. Three millimeter-thick Maximum Intensity Projection (MIP) sections were also obtained. All data was collected in a central database.

### Analysis of Cartilage Calcifications and CT-assessed OA

After a training period, a musculoskeletal radiologist with a 6-year experience and unaware of patient’s details, read all the CT scans. To reduce bias, scans were read in a random order. Calcifications, OA severity in the femoro-tibial compartments and in the proximal tibio-fibular joints were separately read twice with three months apart. Intra-observer reproducibility regarding calcification detection and OA severity was assessed.

Presence of meniscal and hyaline cartilage calcifications of the femoro-tibial compartments and proximal tibio-fibular joints were recorded separately. The results were expressed in terms of femoro-tibial compartments (medial/lateral), knees and subjects. The location of calcifications within the meniscal segments (anterior, middle and posterior) was recorded for each meniscus. The location of hyaline cartilage calcifications was recorded in each medial and lateral femoro-tibial compartments subdivided in thirds laterally (inner, middle, outer) for the tibial plateaus and femoral condyles.

Femoro-tibial compartments and proximal tibio-fibular joints were independently evaluated by CT-scans for OA severity using the Kellgren and Lawrence (KL) grading scale [16]. For each knee and each subject, the highest CT-assessed KL score between the two femoro-tibial compartments (medial/lateral) was selected. CT-assessed OA was considered for KL scores of 2 or more. Femoro-tibial compartments and proximal tibio-fibular joints with a KL score of less than 2 were considered as negative for OA at CT.

### Knee Dissection and Gross Appearance of Cartilage Lesions

The knee dissection and the cartilage macroscopic evaluation were conducted on the 34 left knees by an experimented individual unaware of the results of the CT evaluation. 5 knees had an inappropriate dissection or staining. Knees were thawed at room temperature. Superficial tissues were removed to expose the joint which was then disarticulated. The joint surfaces were rinsed with saline, stained with waterproof India ink (Sanford Rotring, Hamburg, Germany) using a small paintbrush, dried for 10 minutes and rinsed again with saline to remove the ink from the normal surfaces of cartilage so that only the cartilage lesions remained green. The tibial plateaus were then photographed on a fixed stand at a distance of 35 cm with a digital camera (Lumix DMC-FS3, 8.1 Mp, Panasonic, Osaka, Japan). Using an image analyser software (Microvision, Evry, France), we measured separately the surface of plateaus and the stained surface of the plateaus. The evaluation of the cartilage lesion was then expressed as a score equivalent to the ratio of the stained surface on the surface of plateaus.

### Statistical Analysis

Intra-observer reproducibility for detection of calcifications and grading of OA were analysed by the κ statistic. Prevalence estimates for the detection of calcifications and OA were investigated by subject rather than by knee. Subjects were categorized according to the presence or absence of meniscal calcifications (MC), hyaline cartilage calcifications (HCC) or OA in femoro-tibial joints. The prevalence estimates were then computed for MC, HCC and OA. The significance of differences observed for the detection and distribution of calcifications and for the detection of OA was assessed by the Fisher’s exact test, Wilcoxon test or χ^2^.

CT-assessed OA severity was investigated for femoro-tibial compartments and proximal tibio-fibular joints and categorized according to the KL grading scale. The significance of differences observed between medial and lateral compartments was evaluated by the χ^2^ test. The correlation between MC and HCC and between calcifications and CT-assessed OA were investigated by the Fisher’s exact test and by computing the Spearman’s correlation coefficients (r). Statistical analysis were performed using GraphPad Prism^©^ 5.0 for Macintosh. P values <0.05 were considered significant. Confidence intervals were set at 95% (95% CI).

## Results

### Reproducibility

Intraobserver reproducibility was good for the detection of MC (κ = 0.88) as well as HCC in femoro-tibial compartments (κ = 0.92) and proximal tibio-fibular joints (κ = 0.90). Intraobserver reproducibility was substantial for OA scoring, both in femoro-tibial compartments (κ = 0.70) and proximal tibio-fibular joints (κ = 0.72).

### Detection and Distribution of Calcifications

MC were detected in 14/34 (41%) subjects and 23/68 (34%) knees *(* *table 1* *)*. Both knees were involved in 9/14 (64%) and a single knee in 5/14 (36%) subjects. Both menisci were calcified in 16/23 (70%) knees and a single meniscus in 7/23 (30%). A total of 40 menisci were then calcified *(* *table 2* *).* The difference between the medial and lateral femoro-tibial compartments was not significant (p = 0,69). Calcifications involved all three meniscal segments in 25/40 (62.5%), 2 segments in 11/40 (27.5%) and 1 segment only in 4/40 (10%). No case had calcifications involving the middle segment alone. When 2 segments only were calcified, the middle and posterior segments were involved in 8/11 (73%), the anterior and posterior segments in 3/11 (27%). No involvement of the middle and anterior segments alone were observed. HCC were detected in the femoro-tibial joints in 9/34 (26.5%) subjects and 14/68 (21%) knees *(* *table 1* *).* Both knees were involved in 5/9 (56%) and a single knee in 4/9 (44%). Both femoro-tibial compartments were involved in 7/14 (50%) knees and a single compartment in 7/14 (50%). A total of 21 femoro-tibial compartments were then calcified. The difference between the medial and lateral femoro-tibial compartments was not significant (p = 0.32). Tibial plateaus constantly showed calcifications in calcified femoro-tibial compartments (21/21, 100%) *(* *table 3* *).* The calcifications of the tibial plateaus were observed throughout the all thirds of the femoro-tibial compartments in 9/21 (43%) cases, in the inner third alone in 5/21 (24%). No calcifications were observed in associated middle and outer thirds. Femoral condyles demonstrated calcifications in 13/21 (62%) of the calcified femoro-tibial compartments *(* *table 4* *)* with calcifications mainly in all thirds of the femoro-tibial compartments in 5/13 (38%) cases and in the inner third in 3/13 (23%). The calcifications of the tibial plateaus and femoral condyles were significantly correlated (r = 0.89, p = 0.01). HCC were detected in the proximal tibio-fibular joint in 8/34 (24%) subjects and 14/68 (21%) knees *(* *table 1* *)* with bilateral involvement in 6/8 (75%).

**Table 1:** Detection of meniscal calcifications, hyaline cartilage calcifications and CT-assessed osteoarthritis in femoro-tibial and proximal tibio-fibular joints.

	Subjects (%)	Knees (%)	Femoro-tibial compartments	
			Medial (%)	Lateral (%)
**Meniscal calcifications**	**14** (41)	**23** (34)	**19** (47.5)	**21** (52.5)
**Hyaline cartilage calcifications in femoro-tibial joint**	**9** (26.5)	**14** (21)	**8** (38)	**13** (62)
**Hyaline cartilage calcifications in proximal tibio-fibular joint**	**8** (24)	**14** (21)	*N/A*	*N/A*
**CT-assessed osteoarthritis in femoro-tibial joint (KL grade ≥2)**	**15** (44)	**23** (34)	**22** (67)	**11** (33)
**CT-assessed osteoarthritis in proximal tibio-fibular joint (KL grade ≥2)**	**13** (38)	**19** (28)	*N/A*	*N/A*

Detection of meniscal calcifications, hyaline cartilage calcifications and CT-assessed osteoarthritis in femoro-tibial joints and proximal tibio-fibular joints expressed by subjects, knees and femoro-tibial compartments when applicable (*N/A*: not applicable). Osteoarthritis was considered for CT-assessed Kellgren and Lawrence (KL) grade ≥2.

**Table 2:** Distribution of calcifications within the meniscal segments.

Meniscal segments	Medial	Lateral	Total (%)
Anterior	1	1	2 (5)
Middle	0	0	0
Posterior	1	1	2 (5)
Anterior+middle	0	0	0
Anterior+posterior	2	1	3 (7.5)
Middle+posterior	7	1	8 (20)
All segments	8	17	25 (62.5)
**Total**	19	21	40

Distribution of calcifications within the menisci. Each medial or lateral meniscus was divided into anterior, middle and posterior segments for analysis.

**Table 3:** Distribution of calcifications in the hyaline cartilage of the tibial plateaus.

Tibial plateaus (thirds)	Medial	Lateral	Total (%)
Inner	4	1	5 (24)
Middle	0	3	3 (14)
Outer	0	1	1 (5)
Inner+middle	0	1	1 (5)
Inner+outer	1	1	2 (9.5)
Middle+outer	0	0	0
All thirds	3	6	9 (43)
**Total**	8	13	21

Each medial or lateral plateau was divided into inner, middle and outer thirds for analysis.

**Table 4:** Distribution of calcifications in the hyaline cartilage of the femoral condyles.

Femoral condyles (thirds)	Medial	Lateral	Total (%)
Inner	2	1	3 (23)
Middle	0	1	1 (8)
Outer	0	1	1 (8)
Inner+middle	0	1	1 (8)
Inner+outer	1	1	2 (15)
Middle+outer	0	0	0
All thirds	2	3	5 (38)
**Total**	5	8	13

Each condyle was divided into inner, middle and outer thirds for analysis.

### Detection and Severity of CT-assessed OA

Femoro-tibial OA (KL grade ≥2) was present in 15/34 (44%) subjects and 23/68 (34%) knees *(* *table 1* *).* Both knees were involved in 8/15 (53%) and a single knee in 7/15 (47%). OA was present in 33/136 (24%) femoro-tibial compartments. OA was more frequent (p = 0.009) in the medial than in the lateral femoro-tibial compartment with 22/33 (67%) cases *v* 11/33 (33%). OA was also more severe in the medial compartment (χ^2^_df4_ = 15.83, p = 0.003).

OA was observed in the proximal tibio-fibular joints in 13/34 (38%) subjects and 19/68 (28%) knees *(* *table 1* *)* with bilateral involvement in 6/13 (46%).

### Association between MC and HCC

**Figure 1:**
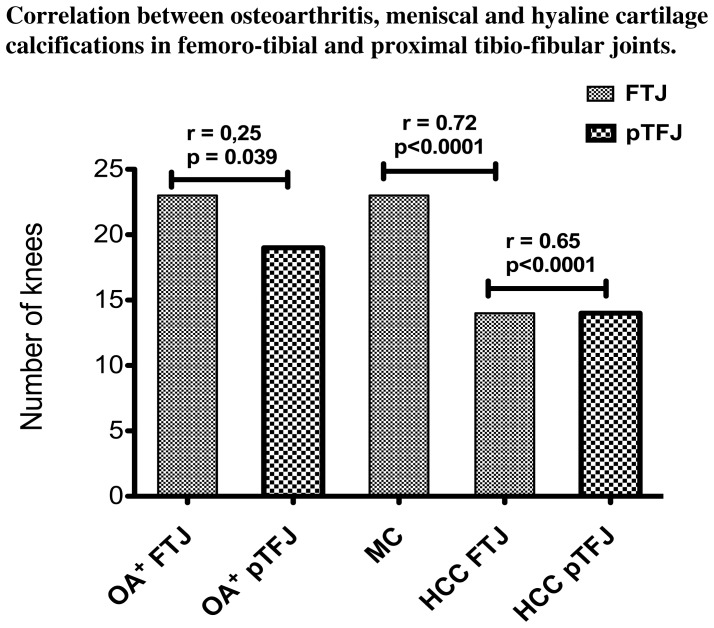
Correlation between CT-assessed osteoarthritis in femoro-tibial joints (OA^+^ FTJ) and proximal tibio-fibular joints (OA^+^ pTFJ). Correlation between meniscal calcifications (MC), hyaline cartilage calcifications in femoro-tibial joints (HCC FTJ) and hyaline cartilage calcifications in proximal tibio-fibular joints (HCC pTFJ). r = Spearman's correlation coefficient, p = value of Fisher's exact test.

A strong correlation between the presence of MC and HCC in the femoro-tibial joint was found (r = 0.72, p<0.0001) *(* *figure 1* *).* HCC were always associated with MC within the same femoro-tibial joint while MC may be isolated. A significant association was also observed between HCC in femoro-tibial joints and in proximal tibio-fibular joints (r = 0.65, p<0.0001) *(* *figure 1* *).* All 14 knees with HCC in proximal tibio-fibular joints also had calcifications in femoro-tibial joints (either MC or HCC in 12 knees or only MC in 2 knees).

### Association of Cartilage Calcifications and CT-assessed OA

HCC were significantly associated with OA in the femoro-tibial compartments (p = 0.004) while MC were not (p = 0.079). No significant association was found either between HCC and OA in the proximal tibio-fibular joint (p = 0.34). A significant association was observed between femoro-tibial and proximal tibio-fibular joints positive for OA at CT (r = 0.25, p = 0.039) *(* *figure 1* *).*

### Gross Appearance of Cartilage

Among the 29 left knees with adequate dissection and staining, MC were detected at CT in 10/29 (34.5%), HCC in 7/29 (24%) and CT-assessed OA were considered in 10/29 (34.5%). The mean surface of cartilage lesion was 35% (range 0.13–0.55, sd 0.11, 95% CI 0.31 to 0.39) *(* *table 5* *)*. The mean surface of cartilage lesion was 36% (range 0.16–0.55, sd 0.13) in knees with MC and 35% (range 0.13–0.50, sd 0.11) in knees without CT-detected MC, with no significant difference (p = 0.79). The mean surface of cartilage lesion was 36% (range 0.13–0.50, sd 0.11) when HCC were detected at CT and 33% (range 0.16–0.55, sd 0.13) when no HCC were detected, with no significant difference (p = 0.6). The mean surface of cartilage lesion was 36% (range 0.18–0.50, sd 0.11) in case of CT-assessed OA and 35% (range 0.13–0.55, sd 0.12) in case of no OA at CT with no significant difference (p = 0.89).

**Table 5:** Mean score of cartilage lesion of the tibial plateaus in knees with/without meniscal calcifications, hyaline cartilage calcifications and CT-assessed osteoarthritis.

	MC+	MC−	HCC+	HCC−	CT-OA+	CT-OA−
**Number of knees (%)**	10 (34.5)	19 (65.5)	7 (24)	22 (76)	10 (34.5)	19 (65.5)
**Mean score of cartilage lesions**	0.36	0.35	0.36	0.33	0.36	0.35

Mean score of cartilage lesion of the tibial plateaus in knees with/without meniscal calcifications (MC+/MC**−**), hyaline cartilage calcifications (HCC+/HCC**−**) and CT-assessed osteoarthritis (CT-OA+/CT-OA**−**). The percentage of knees is calculated out of the 29 left knees analyzed after dissection and ink staining.

## Discussion

**Figure 2:**
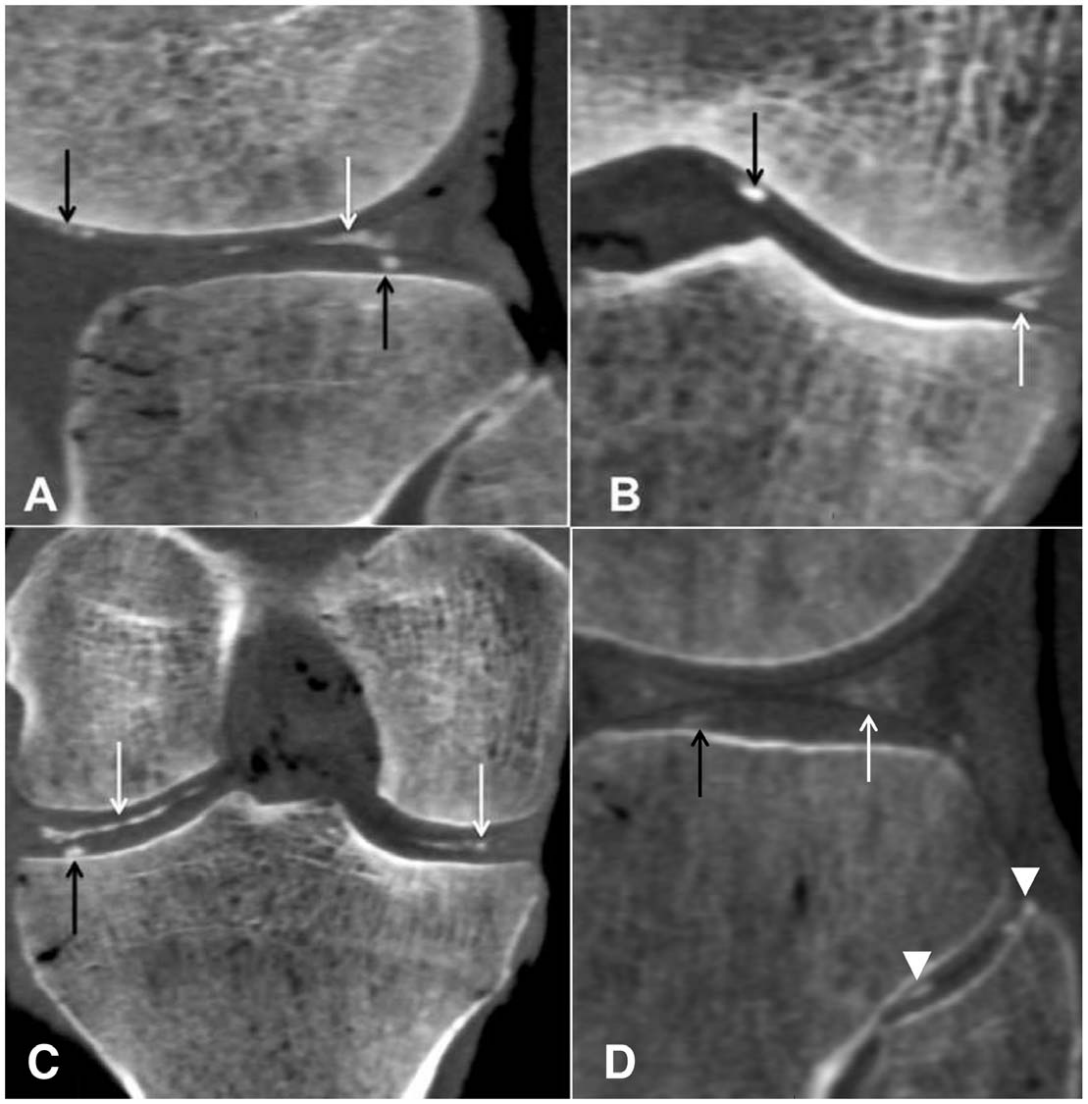
Computed tomography scans of the same knee demonstrating calcifications within the hyaline cartilage (black arrows, arrow heads) and the meniscal segments (white arrows) in the femoro-tibial joint on a sagittal (A) and coronal (B–C) reformations, and in the proximal tibio-fibular joint on a sagittal reformation (D).

**Figure 3:**
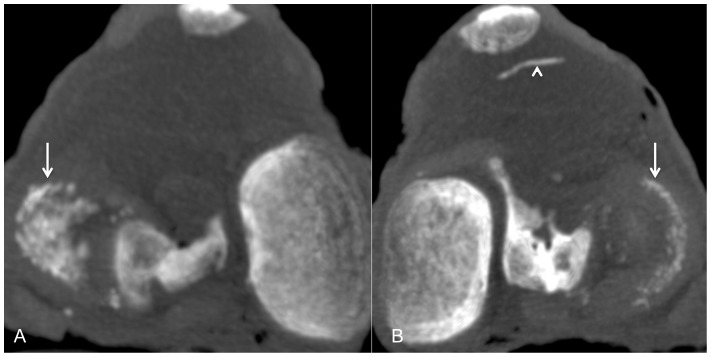
Computed tomographic scans of two different knees on transverse sections (A–B) in Maximum Intensity Projection (MIP, thick section of 3 mm) demonstrating diffuse calcifications within the three meniscal segments (white arrows). Vascular calcifications were observed on the transverse section B (arrow head).

A few studies only have reported the distribution of articular calcifications of the knee, most of them on small series and in comparison with other imaging techniques that were the main topic of the analysis [17]. Moreover and as far as we know, no study in English literature has analysed chondrocalcinosis of the proximal tibio-fibular joint. High-resolution CT-acquisition as well as our experimental conditions (cadaveric knees, absence of motion) provided a fine 3D-analysis of the joint and a precise location of cartilage calcifications *(* *figure 2* *).* The calcifications of the hyaline cartilage were mainly detected throughout the femoro-tibial compartment with a strong correlation of the calcifications between tibial plateaus and femoral condyles. The menisci predominantly showed calcifications throughout their three segments *(* *figure 3* *).* It was also found that articular calcifications tend to have a bilateral distribution and to involve both femoro-tibial compartments in a single knee. Meniscal and hyaline cartilage calcifications were detected in the medial and lateral femoro-tibial compartments with no significant difference. Articular calcifications may also involve both femoro-tibial and proximal tibio-fibular joints. An anatomical communication between the femoro-tibial joint and the proximal tibio-fibular joint does exist in 10 to 27.5% of cases [18], [19]. It may be one explanation to the involvement of both joints since the proximal tibio-fibular joint has limited mobility and undergoes less mechanical stress than the femoro-tibial joint. The loading axis travels effectively mainly through the femoro-tibial joint and there mainly through the medial compartment [20], [21]. These findings may also suggest that calcification of cartilage does not respond only to mechanical stress. The works of Nguyen et al. [3], [22] reports articular calcifications in joint areas bearing less weight such as the femoral intercondylar groove and the lateral femoro-tibial compartment in medial OA. Abreu et al. [23] in their anatomic, radiographic and MRI study of 10 cadaveric knees of elderly individuals also found diffuse calcification in the knee joint in 40% (4/10) of cases, with calcic deposits in menisci and hyaline cartilage but also in other tissues such as cruciate ligaments, popliteus tendon and joint capsule. The strong association we observed between HCC and MC in the femoro-tibial joint and between HCC of both femoro-tibial and proximal tibio-fibular joints may illustrate the tendency of calcifications to be diffuse in the same and adjacent joints. In theory, it may support the hypothesis that cartilage mineralization reflects a altered chondrocyte metabolism or a generalized response of the cartilage to modifications of its biochemical environment. Obviously, this cannot be deducted from these observational results and further investigations including histology are needed to characterize the matrix and cellular changes associated with the cartilage mineralization. Determinants of cartilage calcifications are numerous and reflect a complex process that is not subsequent to joint stress only. Genetic factors [24], aging [25], [26], altered responses to growth factors and inflammatory cytokines [27]–[29] as well as modifications of the extracellular matrix are important factors. More specifically, the imbalance between inorganic pyrophosphate (PPi) and inorganic phosphate (Pi) formation around chondrocytes is a key element that may lead to CPP or BCP crystals within cartilage [30], [31]. Knee joints are particularly interesting to study because of the presence of both hyaline cartilage and fibrocartilage (menisci). In our study, MC appeared more prevalent than HCC. These results were consistent with earlier studies reporting preferential calcifications of the fibrocartilage in comparison with hyaline cartilage [32], [33] **.** We also observed a strong association between HCC and MC. However, HCC were not detected in the absence of MC in the same joint while MC may be isolated in some cases. This may reflect the fact that MC occur before HCC in the femoro-tibial joint.

**Figure 4:**
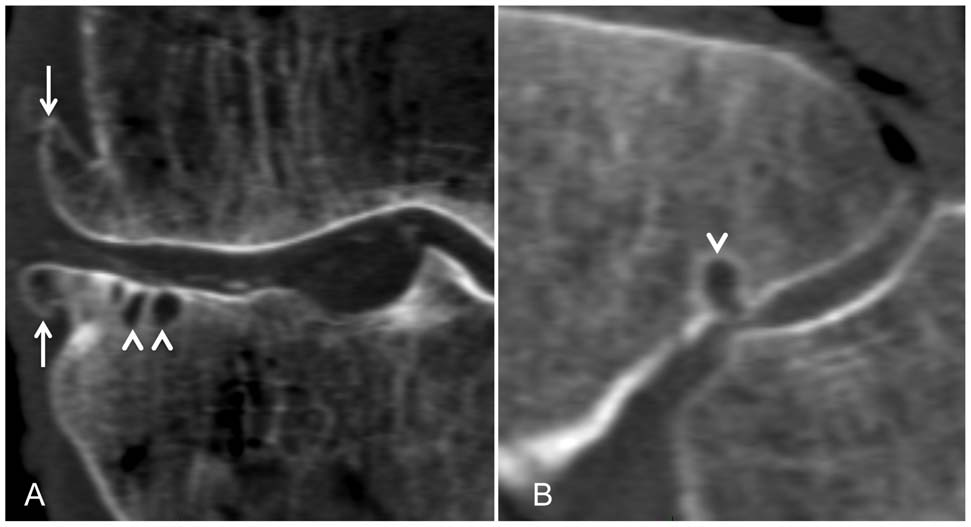
Computed tomographic scans of the same knee showing osteoarthritis in the medial femoro-tibial compartment on a coronal reformation (A) and in the proximal tibio-fibular joint on a sagittal reformation (B). Osteoarthritic changes are consistent with multiple osteophytes (white arrows) and subchondral cysts (arrow heads). Articular calcifications are visible in the femoro-tibial compartment.

The relationship between chondrocalcinosis and OA is complex and remains relevant regarding the pathophysiology of the process leading to cartilage breakdown and OA. The association between OA in femoro-tibial and proximal tibio-fibular joints was interesting to notice as it may reflect a similar trend to OA despite different mechanical stresses. Ozcan and colleagues in their study of the proximal tibio-fibular joints in knees with severe primary femoro-tibial OA also reported an association of degenerative changes between the two joints [34]. We found no significant association between MC and CT-assessed OA whereas HCC were significantly associated with OA. Knowing the protective role of menisci on hyaline cartilage physiology, these finding may argue for a more severe radiographic OA at the stage of HCC rather than at the stage of MC only. We found a slightly higher score of macroscopic lesion in knees with HCC compared with knees without calcifications but no significant difference was found. Moreover, hyaline cartilage lesions were identified in case of articular calcifications (MC or HCC) but also without any detection of calcifications. The CT-assessed OA did not also significantly correlate with the macroscopic evaluation. Given the high mean age of our population and knowing that the age is a main factor of chondrocalcinosis, we could not rule out the fact that the calcifications observed in our study were age-related. Samples are small and the method of ink staining should ideally be completed by further evaluation of the cartilage degradation with routine histology, cartilage thickness measurement and Mankin score [35]. The use of KL grading scale on CT scans to assess OA was also a matter of discussion because of the joint space evaluation being conducted on cadaveric knees and because of the use of CT scans instead of weight bearing radiographs. However, osteophytes and sub-chondral bone changings such as sclerosis and cysts were correctly studied on CT scans *(* *figure 4* *).* Interestingly, Chan and colleagues [36] in their assessment of knee OA comparing radiography, CT and MR showed that CT performance was higher than radiography for the detection of osteophytes in the medial compartment. Moreover, Neame and colleagues [37] in their survey of UK community prevalence of chondrocalcinosis and correlation with OA, evidenced that the only correlation between chondrocalcinosis and OA was through a shared association with osteophytes whereas no significant association between chondrocalcinosis and joint space narrowing was proven. In addition, sub-chondral bone sclerosis was considered for KL grades 3 and 4 and was not changed by our study conditions. Considering the above arguments, it seemed nevertheless informative to assess OA by this grading scale. However, this evaluation remains subjective. As underlined by Neame and colleagues in their study of chondrocalcinosis in a UK community [37], observer bias could not be completely eliminated, as it is difficult to blind for OA when reading for calcifications and vice versa. Interpretation of CT scores of OA and observer bias contributed to the subjectivity of the OA assessment and the decrease of intra-observer reproducibility.

**In conclusion** and as far as we know, the chondrocalcinosis has rarely been studied at CT for the femoro-tibial joint and has never been reported in the literature for the tibio-fibular joint. CT scans of cadaveric knees allowed the detection and description of cartilage calcifications in femoro-tibial and proximal tibio-fibular joints. We report a significant correlation between hyaline and meniscal calcifications in femoro-tibial joints and between calcifications in femoro-tibial and proximal tibio-fibular joints. The detection of hyaline cartilage calcifications correlated with CT-assessed osteoarthritis but no significant difference was found between calcified and non calcified knees at the macroscopic evaluation of the cartilage lesions after dissection. Therefore, further investigations are needed to study the relationship between articular calcification and osteoarthritis changes.
